# DNA Damage, Mutagenesis and Cancer

**DOI:** 10.3390/ijms19040970

**Published:** 2018-03-23

**Authors:** Ashis K. Basu

**Affiliations:** Department of Chemistry, University of Connecticut, Storrs, CT 06269-3060, USA; ashis.basu@uconn.edu; Tel.: +1-860-486-3965

**Keywords:** carcinogenesis, carcinogen, mutagen, metabolism, DNA adduct, tumor, chronic exposure, somatic mutation

## Abstract

A large number of chemicals and several physical agents, such as UV light and γ-radiation, have been associated with the etiology of human cancer. Generation of DNA damage (also known as DNA adducts or lesions) induced by these agents is an important first step in the process of carcinogenesis. Evolutionary processes gave rise to DNA repair tools that are efficient in repairing damaged DNA; yet replication of damaged DNA may take place prior to repair, particularly when they are induced at a high frequency. Damaged DNA replication may lead to gene mutations, which in turn may give rise to altered proteins. Mutations in an oncogene, a tumor-suppressor gene, or a gene that controls the cell cycle can generate a clonal cell population with a distinct advantage in proliferation. Many such events, broadly divided into the stages of initiation, promotion, and progression, which may occur over a long period of time and transpire in the context of chronic exposure to carcinogens, can lead to the induction of human cancer. This is exemplified in the long-term use of tobacco being responsible for an increased risk of lung cancer. This mini-review attempts to summarize this wide area that centers on DNA damage as it relates to the development of human cancer.

## 1. History

In 1761, after use of tobacco for recreation became popular in London, physician John Hill wrote a book entitled “Cautions Against the Immoderate Use of Snuff”. Hill’s observations that tobacco snuff can cause “polypus” (i.e., small vascular growth on the surface of a mucous membrane) led to epidemiology studies nearly 200 years later in 1950s and 1960s, which convincingly established that tobacco smoking causes lung cancer. A few years after Hill’s book was available, in 1775, Sir Percivall Pott of Saint Bartholomew’s Hospital in London published a groundbreaking essay showing that exposure to soot leads to high incidence of scrotal cancer in young men worked as chimney sweeps, which he named the chimney-sweepers’ cancer [1]. This was the first occupational link to cancer. This also was the first association to materials such as soot (a complex mixture of chemicals) to the etiology of cancer. He further hypothesized that the scrotum of young men working as chimney sweeps were particularly susceptible for scrotal cancer later in life, due to their chronic exposure to soot. Sir Percival Pott’s remarkable insight notwithstanding, it took nearly seventy years to pass a law in the UK to protect children from working as chimney sweeps. Perhaps more remarkably, almost 150 years passed when additional studies were attempted on chemical carcinogens, even though an association between certain chemicals and cancer has been reported from time to time. For instance, in 1895 Rehn described the first cases of bladder cancer in German fuchsin dye manufacturing workers. 

An important advance was made in the early 20th century, when Yamagiwa and Ichikawa, two Japanese investigators, developed the first animal assay for carcinogens [2,3]. They repeatedly applied the test compound(s), such as coal tar, on the skin of rabbit ears. Tumors were developed in the experimental animals after a few weeks. Later, rats and mice were found to be better suited for this type of assays [4]. Even though these assays are slow, arduous, and expensive, it continues to be the experimental approach to determine if a compound or a mixture of compounds cause tumorigenesis in mammals. In the 1930s Cook, Kennaway and coworkers were able to isolate and identify benzo[*a*]pyrene (B[*a*]P), a polycyclic aromatic hydrocarbon (PAH), as a potent carcinogen present in soot and coal tar [5,6]. Subsequently, other PAHs were isolated from coal tar and synthetic methods to prepare them were also developed. Over the years, many other groups of compounds and mixtures have been recognized as human carcinogens. Specifically in the 1930s and 1940s, reports of bladder cancers from DuPont and other American dye manufacturers were documented [7,8]. In addition to PAHs (in soot and coal tar) and aromatic amines (present in dyes) [9], numerous other classes of compounds including nitroaromatics [10], asbestos [11], chromium, nickel, and arsenic compounds [12], vinyl chloride [13], aflatoxins [14], diesel exhaust [15], and most notably, tobacco smoke [16], were found to cause cancer. Physical agents like UV light [17] and gamma radiation [18,19] also turned out to be carcinogenic. 

## 2. Metabolic Activation and DNA Damage 

**Figure 1:**
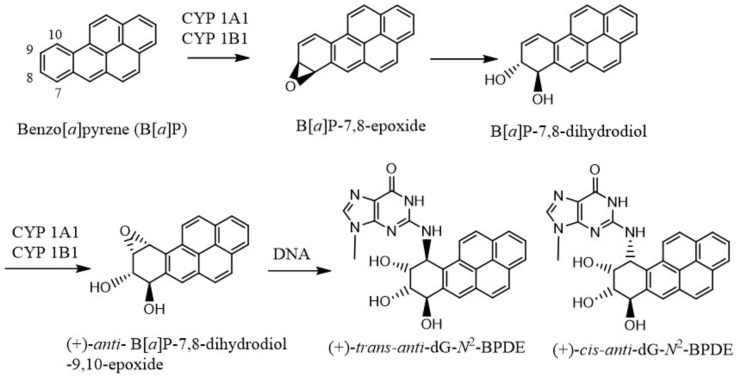
Microsomal metabolic activation of benzo[*a*]pyrene to its most reactive (+)-*anti*-B[*a*]P-7,8-dihydrodiol-9,10-epoxide, which reacts with DNA to form the dG adducts.

In 1950, Boyland proposed that arene oxides are the major metabolites of PAHs that give rise to the phenols, dihydrodiols, and other oxidation products [20]. But the mechanism of the in vivo effects of these carcinogens was little understood until DNA was shown to be the genetic material responsible for coding for all biological processes [21], and the structure of DNA was elucidated by Watson and Crick [22] on the basis of Rosalind Franklin’s unpublished crystal structure of DNA. It became gradually clear that many of the carcinogenic chemicals are metabolically activated to electrophilic species that bind to DNA or cause DNA damage [23,24,25]. Extensive investigations were performed to establish how each carcinogenic agent, either directly or following metabolic changes in their structures, damage DNA or form DNA adducts. As for example, B[*a*]P is converted to 7*S*,8*R*-B[*a*]P oxide by cytochrome P-450 (CYP) 1A1/1B1, which is hydrolyzed by microsomal epoxide hydrolase to form the (−)-7*R*,8*R*-dihydroxydihydro-B[*a*]P [26,27] (Figure 1). This *trans* dihydrodiol is then oxidized again by the same CYP 1A1/1B1 enzymes to form predominantly (+)-*anti*-B[*a*]P-7,8-dihydrodiol-9,10-epoxide, the most mutagenic and tumorigenic metabolite of B[*a*]P. The major adduct formed by this B[*a*]P metabolite is the (+)-*trans-anti*-B[*a*]PDE (Figure 1). 

**Figure 2:**
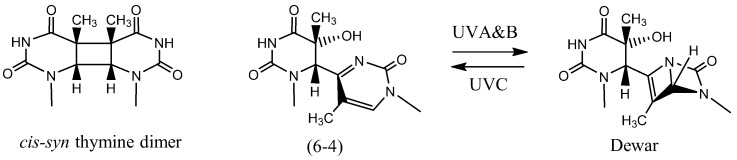
The chemical structures of UV light induced *cis-syn* thymine dimer, pyrimidine(6-4)pyrimidone and Dewar photoproducts formed by two adjacent thymines.

UV light, on the other hand, is an example of a direct acting agent that damages DNA [28,29,30,31], although it also damages DNA indirectly via reactive oxygen species. UV light is considered to be responsible for most skin cancers. UVB (280–320 nm) and UVC (240–280 nm) irradiation form *cis-syn* cyclobutane dimer and pyrimidine(6-4)pyrimidone photoproducts (Figure 2) as the main products in duplex DNA. The chemically stable (6-4) photoproduct may undergo conversion to its Dewar isomer by UVA or UVB light.

**Figure 3:**
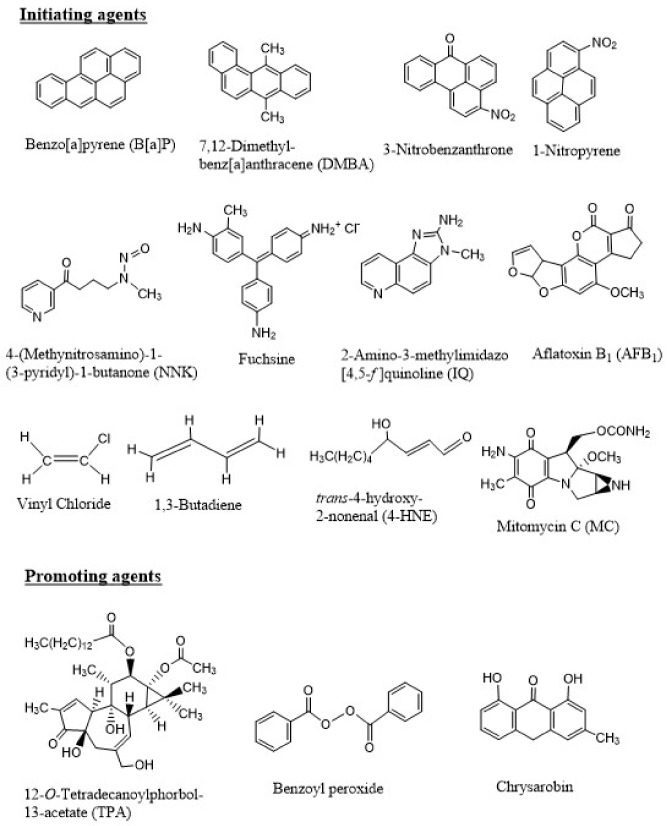
Chemical structures of a few initiating and promoting agents. The initiating agents shown here include polycyclic aromatic hydrocarbons (PAHs) (B[*a*]P and DMBA, present in soot, coal tar, and many environmental mixtures), nitroaromatic compounds (3-nitrobenzanthrone and 1-nitropyrene, present in diesel exhaust), tobacco-specific nitrosamine (NNK, present in tobacco smoke), an amine salt and a magenta dye (fuchsine), aromatic amine (IQ, formed during cooking of meat), a naturally occurring molecule produced by *Aspergillus flavus* (AFB_1_, a food contaminant), industrial chemicals (vinyl chloride and 1,3-butadiene to make the polymer PVC and synthetic rubber, respectively), lipid peroxidation product (4-HNE, produced in cells and tissues of living organisms or in foods during processing or storage), and a chemotherapeutic agent (MC, a toxic drug used to treat upper gastrointestinal cancers). The promoting agents include the phorbol ester (TPA), benzoyl peroxide, and chrysarobin.

Similar to B[*a*]P, metabolism and DNA binding by a large number of chemical carcinogens have been reported. Figure 3 shows the chemical structures of a few of these carcinogenic compounds, which include PAHs, nitroaromatic compounds, aromatic amines, natural products, industrial chemicals, and a chemotherapeutic agent that also induces secondary tumor. 

## 3. Multi-Step Process of Cancer

As early as in the 1940s, it became apparent that the process of carcinogenesis involves at least two distinct steps. In 1944, Mottram showed that a single application of a carcinogen, such as B[*a*]P, followed by multiple applications of an “irritant”, such as croton oil, induce tumors in animals [32]. Berenblum and Shuvik followed up this study with application of either B[*a*]P or 7,12-dimethylbenz[*a*]anthracene (DMBA) and croton oil, and demonstrated that croton oil, a non-carcinogen, had no effect alone, but when applied after even a single dose of either B[*a*]P or DMBA on mouse skin, tumors were developed [32]. These results led to the hypothesis of “initiation” (result of application of the carcinogen like B[*a*]P) followed by “promotion” (caused by croton oil). Later, croton oil was shown to contain the phorbol ester, 12-*O*-tetradecanoylphorbol-13-acetate (TPA), as the active ingredient that is responsible for the promotion phase in carcinogenesis [33]. Additional tumor promoters, including benzoyl peroxide, okadaic acid, chrysarobin, have been identified (Figure 3). 

**Figure 4:**
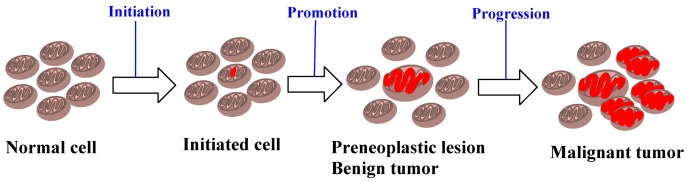
A brief depiction of initiation, promotion, and progression in the process of carcinogenesis.

There are several fundamental differences between these two stages (and the agents that trigger these processes) [34,35,36,37]. An initiating agent is also a “complete carcinogen”, since either repeated exposure in small dosage or a single large exposure to such agents lead to carcinogenesis, whereas a promoting agent is not carcinogenic alone. The effect of an initiating agent, in addition, is irreversible and additive, in contrast to the reversible action of a promoting agent at the early stages. The initiating agents furthermore become electrophilic after metabolic activation, and bind to cellular macromolecules such as DNA, while there is no evidence of covalent binding by the promoting agents. The initiating agents are mutagenic and, as a result, quite a few short-term assays have been developed [38,39,40,41], whereas the promoting agents are not mutagenic. Experiments in rodents on the two-stage model, however, showed that mainly benign tumors were developed by tumor promoters [42]. It became gradually accepted that carcinogenesis involves multi-stages, which include initiation, promotion, and malignant progression, when benign neoplasms become malignant and invasive lesions [43] (Figure 4). 

Discovery of oncogenes and tumor suppressor genes added to the concept that carcinogenesis is a multi-step process [43,44]. Notably, continuous oxidative stress and chronic inflammation sustain each other, leading to neoplasm, and promote tumor progression. Inflammation has been associated with the development of cancer, and inflammatory mediators, like cytokines, chemokines, and eicosanoids, have been shown to stimulate the proliferation of both untransformed and tumor cells [45]. Certain initiating agents, such as UV light and tobacco smoke also exhibit strong tumor promoting activity. 

Most of our understanding of tumor promotion comes from experiments performed on mouse skin [46]. The promotion stage in carcinogenesis induces a number of epigenetic changes, including proliferation of epidermal cells and activation of ornithine decarboxylase that leads to synthesis of polyamines [47,48,49]. Overall, the promotion stage is characterized by hyperplasia, that leads the initiated cells to form papillomas. Strong tumor promoters, such as the phorbol esters, activate membrane receptors like protein kinase C [50]. Activation of protein kinase C phosphorylation of critical proteins is considered an important event in skin tumor promotion. Several other tumor promotors, including benzoyl peroxide, appear to involve free-radical mechanisms, which indirectly lead to phosphorylation of certain proteins [51]. Tumor promotion is also characterized by clastogenic effect and genetic instability, resulting in chromosomal alterations. Consequently, tumor promotion includes a series of complicated epigenetic steps leading to formation of papillomas. Tumor promotion can also be induced by tumor necrosis factor-α (TNF-α) and TNF-α-inducing protein (Tipα) of *Helicobacter pylori* stimulates progression phase [52]. Recent studies on human cancer development includes upregulation of TNF-α and activation of NF-κB, an important transcription factor [52].

## 4. DNA Damage and DNA Repair

**Figure 5:**
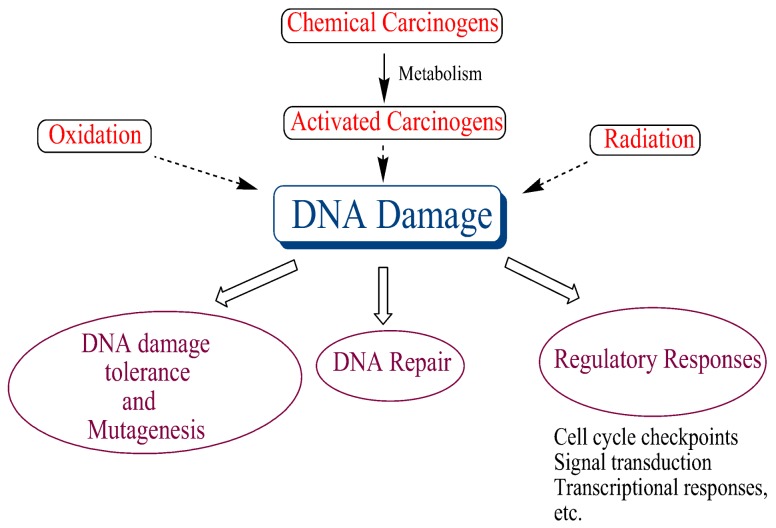
DNA damage plays a central role in many biological processes linked to cancer.

DNA damage occurs continuously in all organisms via a number of endogenous and exogenous factors, and it seems to play a central role in many biological processes, ultimately leading to cancer (Figure 5). Hence, robust DNA repair systems, which repair this damage, have evolved to maintain genomic integrity. The importance of DNA repair was underscored by conferring the Nobel Prize in Chemistry in 2015 to Tomas Lindahl, Paul Modrich, and Aziz Sancar for mapping, at a molecular level, how cells repair damaged DNA and protect the genetic information. There are a number excellent reviews on DNA repair, which summarize this rapidly evolving field [53,54,55,56,57].

DNA replication occurs during the S (synthetic) phase of cell cycle, which is preceded by the G1 (Gap 1) phase. The nuclear division occurs in the M (mitosis) phase, which takes place after the G2 phase. The differentiated cells at the G0 phase do not proliferate, whereas the G1, S, and G2 phases of a proliferating cell constitute the time lapse between two consecutive mitoses. The progression of a cell during cell cycle is regulated by cyclin dependent kinase in order to avoid the initiation of a cell cycle before the preceding one is completed. DNA damage interferes with the cell cycle, and therefore, there are checkpoint proteins that delay cell cycle progression providing the necessary time for DNA repair. If the DNA damage exceeds the capability of repair, pathways to trigger cell death are activated by apoptosis. The checkpoint pathways accordingly play an integral role in DNA damage response, and dysfunction of these pathways are important for the pathogenesis of malignant cells [58]. 

## 5. Relationship between DNA Adducts and Tumor Incidence

Carcinogens and mutagens usually generate multiple DNA adducts, and it was shown that certain adducts are biologically more relevant than others. Many diseases in humans are the result of specific genetic mutations. Therefore, DNA adducts or lesions that lead to mutations became the focus of many studies. As for example, the predominant mutation induced by most methylating and ethylating agents are G:C→A:T transitions induced by *O*^6^-alkylguanine, even though the major adduct is formed at the N7-position of guanine [59]. 

Characterization of a quantitative relationship between DNA adduct levels and tumor incidences in rats and mice was attempted by Ottender and Lutz [60]. Of the 27 different chemicals investigated, the range of carcinogenic potency of structurally different DNA adducts is typically within 2 orders of magnitude. In the rat, for instance, 53 adducts per 10^8^ nucleotides for the aflatoxin B1 to 2082 adducts per 10^8^ nucleotides for dimethylnitrosamine relate to the normalized 50% level of liver tumor incidences, suggesting that the aflatoxin–DNA adducts are 40 times more potent than the adducts formed by dimethylnitrosamine for inducing hepatocellular carcinoma. 

## 6. Damaged DNA Replication

DNA replication causes mutations and DNA damage, or DNA adducts increases the rate of error-prone replication [61]. However, each DNA damage or adduct has a unique mutational signature, which is directly related to the identity of the DNA polymerase that bypass it and the mechanism of its nucleotide insertion and extension [61]. 

A human cell contains at least 17 different DNA polymerases. The DNA polymerases belong to seven families (A, B, C, D, X, Y, and RT) [62,63], of which the C family enzymes were only found in prokaryotes. In eukaryotes, the B-family enzymes pol ε and pol δ carry out a large fraction of nuclear DNA replication, whereas pol α of the same family performs initiation and priming. These three polymerases are essential for DNA replication in eukaryotes. In the current model of DNA replication, pol ε carries out majority of leading strand DNA replication of the undamaged genome, whereas pol δ primarily replicates the lagging strand. But this model has recently been challenged, and data supporting involvement of pol δ in both leading and lagging strand replication have been presented [64,65,66]. It is noteworthy that these important DNA polymerases are inefficient in bypassing most bulky or distorting DNA damages, such as the ones induced by PAHs and UV light. 

The discovery of translesion synthesis (TLS) DNA polymerases in the 1990s and the study of their catalytic and non-catalytic roles in damaged DNA replication provided much of our current understanding of DNA adduct or lesion bypass [63]. Lesion bypass is carried out primarily by the Y-family polymerases. But X- and B-family polymerases are also involved in many cases.

TLS of various types of DNA damage have been conducted by genetic studies in repair and replication competent cells, by in vitro experiments using purified DNA polymerases and accessory proteins, and by structural and computational studies. The mechanistic information gathered from these studies is critical to understand the mechanism of mutagenesis, the underlying process for the development of cancer. These fundamental studies are now allowing therapeutic application, as inhibiting the activity of some of the TLS polymerases may enhance the effect of an antitumor agent. 

## 7. Epidemiology

At the international cancer congress held in Tokyo in 1966, Sir Alexander Haddow, the President of international union against cancer, pronounced: “We are impressed by the probability that a much higher proportion of human cancer than we had ever recently suspected—perhaps amounting to as much as 80 percent—may be due to environmental causes” [67]. These remarks from an eminent cancer researcher are significant, because it suggests that most human cancers are preventable. The most common preventable risk factors for cancer are tobacco smoking, diet (low in fruits and vegetable and high in fatty foods, red meats, etc.), obesity, and alcohol [68,69,70,71,72,73]. Cancer rate also increases with age, but age-related cancer patterns are fairly complex. 

Epidemiology showing the definitive link between tobacco smoke and cancer was a noteworthy achievement in the United States, and the Surgeon General’s Report in 1964 had a significant positive effect on public health in this country. The smoking prevalence in males decreased by about 60%, while prevalence in females diminished by about 50% [74]. As a result, lung cancer mortality and other tobacco-related diseases continue to decrease. These facts reiterate the importance of tobacco control in prevention of cancer and other diseases [16,75,76,77].

Epidemiology of skin cancer has also been enlightening [78]. One in every three cancers diagnosed is a skin cancer, and one in every five Americans will develop skin cancer in their lifetime. Melanoma and nonmelanoma skin cancer (NMSC) are the most common types of cancer mainly in the white populations. Both types of tumors show an increasing incidence rate worldwide, but a stable or decreasing mortality rate, presumably due to earlier diagnosis and better treatments. NMSC is the most common cancer in fair-skinned individuals, which causes significant morbidity. The rising incidence rates of NMSC are believed to be triggered by a combination of increased exposure to direct UV rays or UV in sunlight, increased longevity, ozone depletion, genetics, and in a limited number of cases, immune suppression. 

## 8. Mutation and Cancer

Several types of cancers are the result of at least a few mutations in critical genes [79,80]. The somatic mutation theory (SMT) of cancer, the most prevalent model, proposes that cancer is caused by mutation(s) in the body cells (as opposed to germ cells), especially nonlethal mutations associated with increased proliferation of the mutant cells. The SMT hypothesis originated from Theodore Boveri’s postulate in 1914 that a combination of chromosomal defects could result in cancer [81]. After Watson and Crick’s discovery of the structure of DNA that also implied that DNA contains the genetic information, in 1953, Carl O. Nordling proposed that several mutated genes may lead to cancer [82]. Ashley suggested that cancer may occur as a result of three to seven mutations [83]. Alfred Knudson modified Ashley’s proposal, based on his observations of a number of retinoblastoma cases, proposing that cancer is the result of accumulated mutations to a cell’s DNA, which could be as little as two hits [84]. The two-hit model proposes that dominantly inherited predisposition to cancer requires a germline mutation, while tumorigenesis necessitates a second somatic mutation. For colorectal carcinoma, Fearon and Vogelstein suggested that four to five gene mutations are necessary for the development of malignant tumor, and the accumulation of the mutations, rather than their specific order, is the critical determinant of tumorigenesis [85]. More recently, these mutations have been referred to as “driver” mutations conferring growth advantage to the cells [79]. In humans, more than 350 mutated genes that are implicated in the development of cancer have been identified. A large-scale sequencing study has shown that most somatic mutations in cancer cells are “passengers” that do not cause tumorigenesis, whereas 120 of the 518 genes screened (~23%) carry a “driver” mutation, which can function as cancer genes. Similar conclusions have been reached in other studies [79,86]. The basic premise of SMT, however, has been challenged from time to time [87]. 

Genes that contribute to cancer include oncogenes and tumor suppressor genes. Oncogenes change a normal healthy cell into a cancerous cell. Examples include the *ras* family of genes and *HER2*. The *ras* genes produce proteins engaged in cell communication pathways, cell growth, and cell death, whereas *HER2* makes specialized proteins controlling cell growth, and spread notably in breast and ovarian cancer cells. DNA adduct-induced mutations in the *ras* gene, at the activating codons 12, 13, 59, and 61, are considered to be of note. Aflatoxin B_1_ (AFB_1_) causes G·C→A·T or G·C→T·A substitutions at codon 12 in experimental animals [88]. Analyses of lung tumors in A/J mice by the tobacco-specific nitrosamine 4-(methylnitrosamino)-1-(3-pyridyl)-1-butanone (NNK) and related compounds showed high frequency of G→A mutations (GGT to GAT) in codon 12 [89]. By contrast, tumor suppressor genes protect a cell from becoming cancerous. The tumor suppressor proteins control cell growth by monitoring cell division, repairing base mismatches in DNA, and controlling cell death (apoptosis). Examples of tumor suppressor genes include *p53*, *BRCA1*, and *BRCA2*. More than 50% of human cancers are characterized by mutations in the *p53* gene, and most *p53* gene mutations are not hereditary. Germline mutations in *BRCA1* or *BRCA2* gene increases a woman’s risk of hereditary breast and ovarian cancer. A convincing relationship between a chemical and *p53* mutation in human cancer has been shown in geographical areas where AFB_1_-derived liver cancers accompanied unusually high frequency of G·C→T·A mutations at the third base of codon 249 of the *p53* gene [90]. Also, a human liver cell line following exposure to AFB_1_ showed the same mutation at the third base of *p53* codon 249 [91]. Likewise, for lung cancer cases of smokers, ~40% of the mutations involved G→T transversions, and more than 90% of them are on a guanine on the non-transcribed strand [90]. Major hotspots are observed at codons 157, 248, and 273. Even though codon 157 is unique to lung cancer, the other two are hotspots for mutations in many other cancers, usually detected as transitions at these CpG sequences, whereas in lung cancers, G→T transversions are the most common mutations [92]. Pfeifer and colleagues have claimed that sequence specificity of G→T transversions in lung tumors is consistent with a direct mutagenic action of PAH compounds, such as B[*a*]P present in cigarette smoke [93]. In addition to the cancers induced by exogenous agents, few hereditary cancers, which include retinoblastoma and Li-Fraumeni syndrome, involve germline mutations in tumor suppressor genes [94,95]. 

Human tumors are largely heterogeneous. Loeb and coworkers suggest that this heterogeneity results from a mutator phenotype. They hypothesized that increased mutation rates are essential to account for the large number of mutations observed in cancer cells [96,97]. Consequently, an initial mutator mutation triggers additional mutations, including mutations in genes that maintain genetic stability, starting a cascade of mutations throughout the genome. Several types of cancers exhibit mutator phenotype resulting from mutations at loci responsible for DNA mismatch repair [98]. It was also proposed that *p53* mutations might give rise to mutator phenotype, because p53 is a gatekeeper of DNA damage responses [99]. However, others believe that a mutator phenotype is not necessary for tumor initiation and progression, in spite of the fact that some tumors may acquire it during tumorigenesis [100]. 

In one of the most highly cited articles, entitled “Hallmarks of Cancer”, in the year 2000, Hanahan and Weinberg suggested that the complexity of cancer can be summarized in six hallmarks that enable normal cells to turn tumorigenic and ultimately malignant [101]. These hallmarks are as follows: (1) self-sufficiency in growth signals, implying the ability of tumor cells to grow in the absence of the signals that allow them to grow, (2) insensitivity to anti-growth signals, i.e., they resist the signals to stop growth, (3) evading apoptosis, i.e., they resist their programmed death, (4) limitless replicative potential, so that they can multiply indefinitely, (5) sustained angiogenesis, i.e., they stimulate the blood vessel growth in order to supply nutrients to the tumor cells, and (6) tissue invasion and metastases, i.e., they invade surrounding tissues and spread to distant sites. However, Lazebnik pointed out that hallmarks 1–5 are also the characteristics of benign tumors [102]. In an update of the *Hallmark* paper, in 2011, Hanahan and Weinberg proposed four additional hallmarks: (1) abnormal metabolic pathways, (2) evading the immune system, (3) genome instability, and (4) inflammation [103]. In principle, the cancer phenotypes proposed as hallmarks are based on the SMT and its cell-centered variants. Others, though, argued that cancer is a tissue-level disease and cataloguing such cellular-level hallmarks are misleading [104].

## 9. Conclusions

A detailed understanding of multi-stage carcinogenesis is important for both the treatment and prevention of cancer. This area of research, for the last fifty years, has provided us a great deal of mechanistic information on initiation, promotion, and progression, the three main steps leading to cancer. Consequently, many types of cancer deaths have been reduced in the USA over the last two decades, to an overall reduction of 23%, and more than 1.7 million cancer deaths were averted [105]. In spite of this progress, cancer is still the leading cause of death for much of the US population. Likewise, there has been significant reduction in several European countries. Unfortunately, progress has been limited in many other countries, due to the lack of adequate cancer diagnosis and limited medical treatment capabilities [106,107]. In fact, more than 60% of the world’s new cancer cases take place in Africa, Asia, and Central and South America, and 70% of the world’s cancer deaths occur in these continents. Therefore, it is imperative to continue further studies on the mechanism of carcinogenesis with the objective of prevention, treatment, as well as developing new strategies to combat this deadly disease. 

## Abbreviations

PAH	polycyclic aromatic hydrocarbon
B[*a*]P	benzo[*a*]pyrene
CYP	cytochrome P-450
UV	ultraviolet
TPA	12-*O*-tetradecanoylphorbol-13-acetate
TNF-α	tumor necrosis factor-α
TLS	translesion synthesis
NMSC	nonmelanoma skin cancer
SMT	somatic mutation theory
